# Application of an L-shaped anterolateral thigh flap in reconstruction after hemiglossectomy

**DOI:** 10.1186/s12893-022-01473-7

**Published:** 2022-01-29

**Authors:** Xi Rui, Zixian Huang, Jiyuan Zuo, Yan Wang, Qixiang Liang, Tingting Jin, Jianguang Wang, Shaohai Chang, Zhiquan Huang

**Affiliations:** 1 Guangdong Provincial Key Laboratory of Malignant Tumor Epigenetics and Gene Regulation, Sun Yat-Sen Memorial Hospital, Sun Yat-Sen University, Guangzhou, 510120 China; 2 Department of Oral and Maxillofacial Surgery, Sun Yat-Sen Memorial Hospital, Sun Yat-Sen University, 107th Yanjiang Xi Road, Guangzhou, 510120 Guangdong China; 3 Guanghua School of Stomatology, Sun Yat-Sen University, Guangzhou, China; 4 Department of Stomatology, The Third Affiliated Hospital, Sun Yat-Sen University, Guangzhou, 510630 Guangdong China; 5 Department of Stomatology, Sun Yat-Sen Memorial Hospital, Sun Yat-Sen University, 107th Yanjiang Xi Road, Guangzhou, 510120 Guangdong China

**Keywords:** Anterolateral thigh perforator flap, Tongue cancer, Hemiglossectomy, Tongue reconstruction, Postoperative function

## Abstract

**Objective:**

Tongue defect reconstruction is one of the key components of tongue cancer surgery. In this study, we used an L-shaped flap design adopted as a simple and efficient method to repair tongue defects after hemiglossectomy. Furthermore, we evaluated and contrasted the clinical effects of two methods, the L-shaped and traditional methods.

**Study design:**

Fifteen patients in the L-shaped group and 20 patients in the traditional group were evaluated and compared in terms of postoperative complications, dysphagia, language function and appearance satisfaction.

**Results:**

The results (Table 1) showed that there were 2 cases of donor area invalid traumas, and 2 patients had scar hyperplasia in the traditional group. The degree of global and functional dysphagia of the L-shaped group (2.60 ± 0.29 and 11.47 ± 1.38) was lower than that of the traditional group (3.55 ± 0.29 and 15.75 ± 1.22) (P < 0.05). In the language evaluation, the traditional group (3.20 ± 0.26) had lower scores than the L-shaped group (4.13 ± 0.30) (P < 0.05).

**Conclusion:**

The L-shaped ALTP flap is a simple and efficient modification of ALTP, that can be used for half-tongue repair after radical operations for tongue cancer. It has better performance in the recovery of dysphagia and language function than the traditional ALTP flap.

**Supplementary Information:**

The online version contains supplementary material available at 10.1186/s12893-022-01473-7.

## Introduction

The tongue is the most common site of oral cancer, and most oral cancer cases are squamous cell carcinoma, which has a high degree of malignancy [1], a high rate of local recurrence, and commonly has early lymph node metastasis [2, 3]. Extended resection is one of the standard types of surgical treatment [4]. However, the tongue plays an important role in swallowing and auxiliary pronunciation. Dysphagia and dysarthria can cause mental depression and malnutrition in patients with tongue cancer [5]. To avoid postoperative language and mastication dysfunctions caused by tongue defects and to improve the patients’ quality of life, it is very important to repair defects after radical operations for tongue cancer. Free flap transplantation is currently the most effective way to reconstruct the tongue after resection.

Free forearm flaps, anterolateral thigh free flaps, and rectus abdominal free flaps are commonly used in reconstructive surgery [6, 7]. However, there are still many problems; for example, the forearm flap lacks muscle, so it may not be able to completely fill large defects, may lead to a dead cavity, and to a certain extent, may adversely affect the shape and function of the forearm [8, 9].

The anterolateral thigh flap (ALTP) has been used widely in the reconstruction of tongues after radical operations for tongue cancer. However, some patients treated with the traditional rectangle shaped ALTP had cases of mismatching, flap necrosis, and donor site infection, and had poor postoperative function and appearance dissatisfaction.

To reduce the occurrence of complications and obtain better postoperative reconstruction results, we designed an L-shaped flap based on the position of the perforators for patients with half-tongue defects. The flap is selected based on the condition of the tongue defect so that the free flap is used to its full extent to restore the original shape of the tongue and preserve the tissue in the donor area. Regardless of whether the tip of the tongue is preserved, the L-shaped flap shows good performance in tongue reconstruction. Furthermore, this study compared the performance of the L-shaped flap and the traditional rectangle-shaped anterolateral thigh flap in tongue reconstruction after hemiglossectomy.

## Methods and patients

This research was conducted in accordance with international guidelines and the ethical standards outlined in the Declaration of Helsinki. It was approved by the Sun Yat-sen Memorial Hospital Institutional Review Board, and all participants signed an informed consent form.

### General information

From January 2017 to June 2019, 35 patients with tongue squamous cell carcinoma who underwent hemiglossectomy and tongue repair were selected as the study subjects. No tumours infiltrated the mandible in any of the patients. The patients were divided into two groups according to the type of flap used in half-tongue repair: the L-shaped ALTP flap group (L-shaped group) and the traditional ALTP group (traditional group). There were 15 patients in the L-shaped group, including 9 men and 6 women, with an average age of 47.73 years (29–68). There were 20 patients in the traditional group, including 15 men and 5 women, with an average age of 54.05 years (29–79). All patients underwent an evaluation for TNM staging according to the seventh edition of the American Joint Committee on Cancer (AJCC). To reduce the risk of postoperative recurrence, we performed a complete resection of all tumours.

### Design of the L-shaped flap

To retain as much tissue in the donor area as possible while providing a sufficient volume for the tongue tip and a sufficiently wide tongue base, the flap was designed based on the position of the perforator and the status of the tongue.

**Fig. 1:**
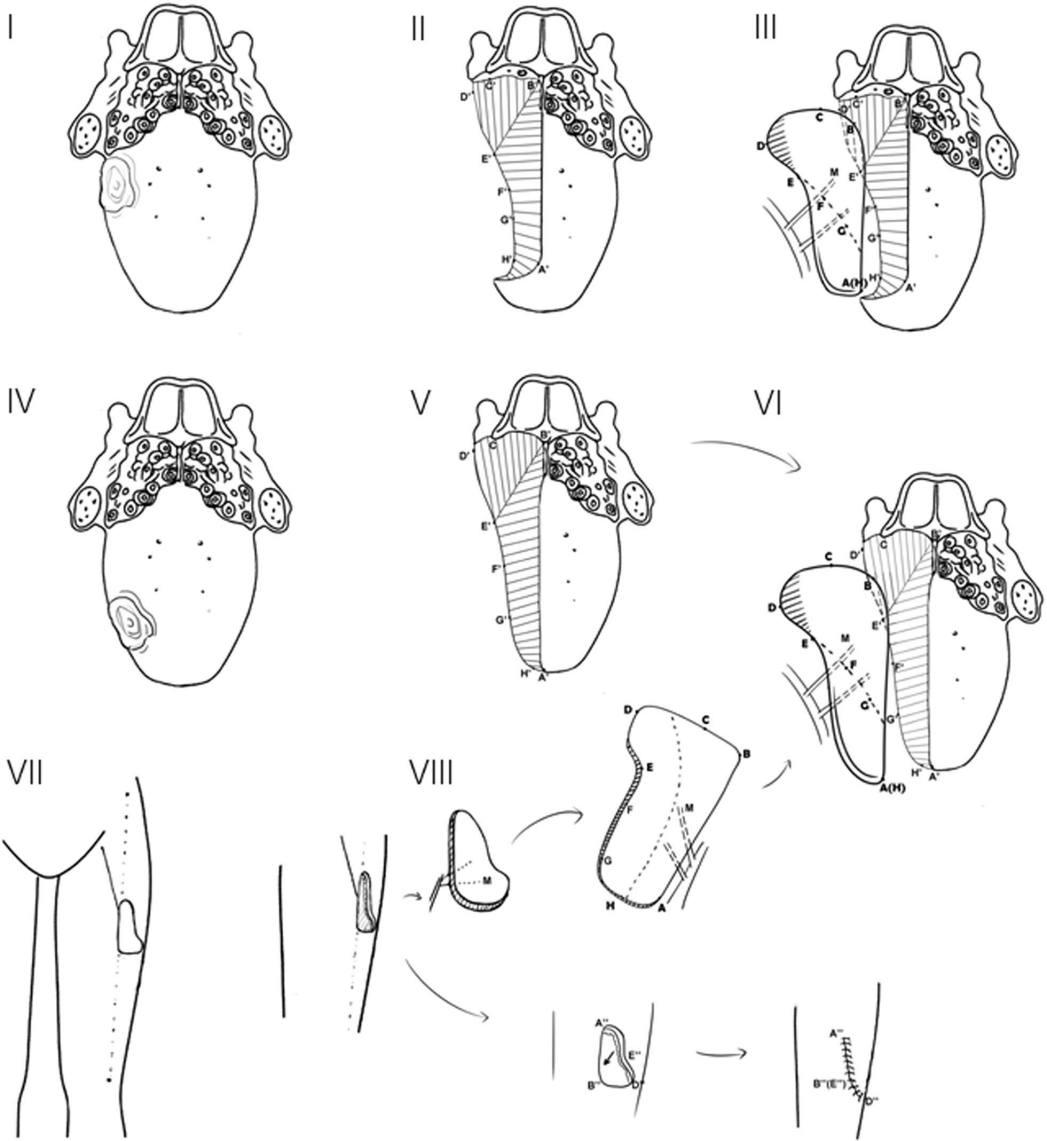
Design of the L-shaped ALTP. Schematic diagram of the flap repair after hemiglossectomy with or without the tip of the tongue preserved (I-III Tongue tippreserving hemiglossectomy; IV-VI Standard hemiglossectomy; VII-VIII Donor area suture); the landmarks on the flap correspond to the defect. The landmarks of the tongue defect are as follows:** A** the most anterior point of the defect on the dorsum of the tongue;** B** foramen caecum of the tongue;** C** the posterolateral point of the tongue;** D** the outer edge of the pharyngeal defect;** E** the junction of the lingual gingiva of the posterior teeth and floor of the mouth;** F** the posterior point of the ventral tongue;** G** the frenum linguae; and** H** the anterior point of the defect on the ventral tongue (I–III tongue tip preserving hemilingual excision repair; IV–VI standard hemilingual excision repair; VII–VIII donor site suture)

After anaesthesia was induced, the tongue was pulled to the position of the lower incisor for measurement. The following sites were labelled and measured: A: the most anterior point of the defect on the dorsum of the tongue; B: the foramen caecum of the tongue; C: the posterolateral point of the tongue; D: the outer edge of the pharyngeal defect; E to F: the junction of the lingual gingiva of the posterior teeth and the floor of the mouth; F: the posterior point of the ventral tongue; G: the frenum linguae; and H: the anterior point of defect on the ventral tongue. Preoperative vascular exploration and perforating branch localization were performed. The surface projection point of the perforator was marked as “M”. The donor flap was clipped according to the above measurements with “M” as the centre (Fig. 1).

### Surgical technique

**Fig. 2:**
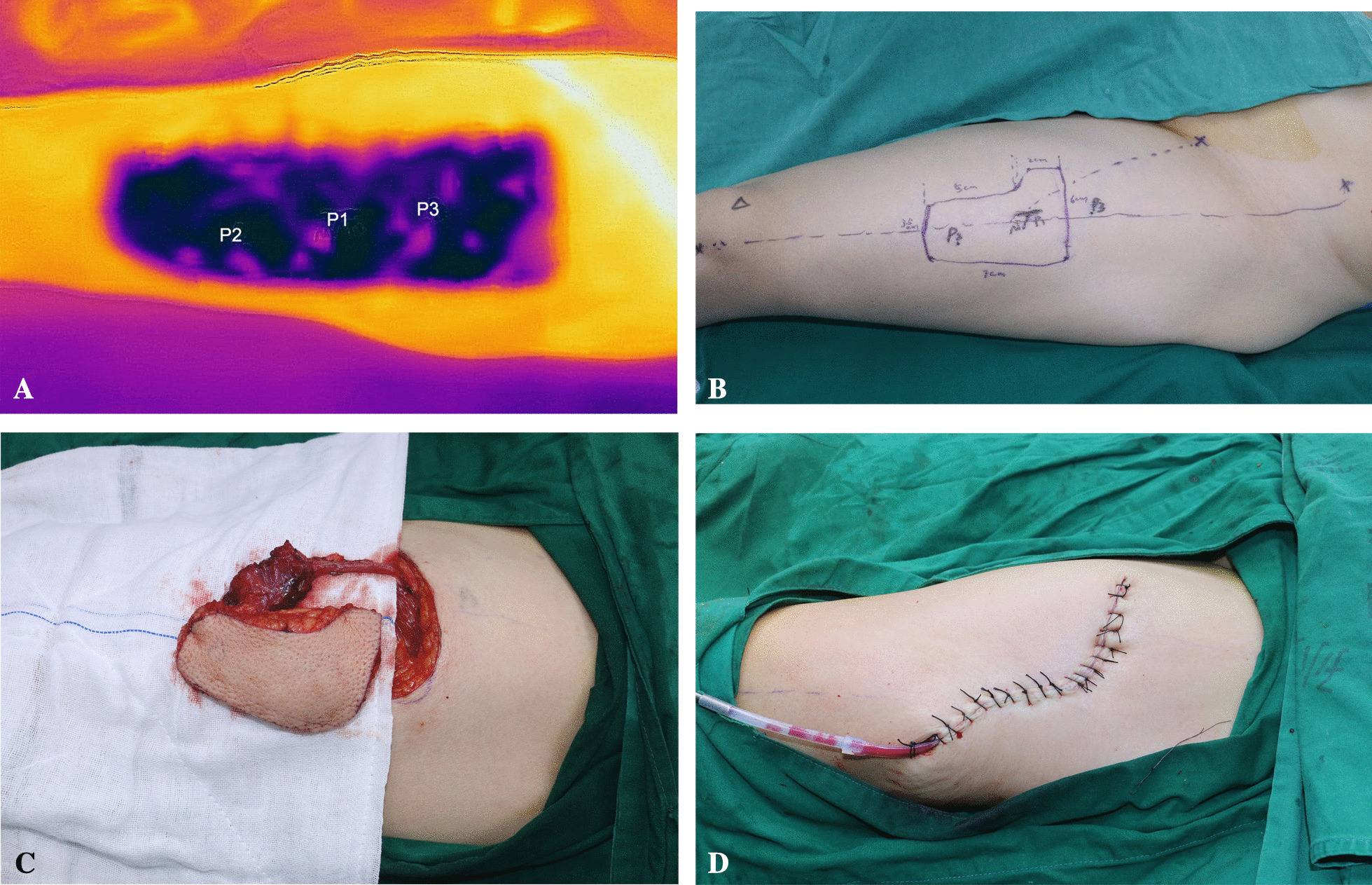
L-shaped ALTP preparation. **A** The perforating branches in the donor area were examined. **B** Based on the perforators, an L-shaped flap was designed, and at least one perforator was retained. **C** The flap was prepared. **D** The donor area of the thigh was sutured, and a drainage tube was left in place

Before the operation, the blood vessels, especially the perforating branch in the donor area, were mapped using Doppler ultrasound or a smartphone-compatible thermal imaging camera [10] (Additional file 1: Video. S1). According to the examination results, a half-tongue repair scheme was designed (Fig. 2, Additional file 2: Fig. S1). All patients underwent extended local excision and ipsilateral neck lymphadenectomy. Moreover, preserving the tip of the tongue is very important for the recovery of postoperative function. For some tumours that did not exceed the midline and occurred in the posterior 1/3 of the tongue, hemiglossectomy with the tongue tip preserved was performed. For tumours that did not exceed the midline and occurred in the anterior 2/3 of the tongue, standard hemiglossectomy was performed (Fig. 1).

The vessels of the recipient area were reserved according to the conditions during the operation. Skin flaps with sizes and shapes corresponding to the plan made before the operation and the condition of the tongue defect were harvested. The incision in the donor area was closed and sutured directly (Fig. 2, Additional file 3: Fig. S2). The prepared flap was transferred to the defect area of the tongue and aligned according to each marked point (Fig. 1); then, the vessels were anastomosed. The superior thyroid artery or the external maxillary artery was anastomosed with the lateral circumflex artery. The facial vein and/or the external carotid artery were anastomosed with the lateral circumflex artery. Then, the remaining tongue tissue and mucous membrane were sutured. The operation and flap removal time in all patients were recorded.

Blood circulation in the flap was closely observed for 24 h after the operation, and vascular exploration was performed in cases of vascular crisis. In addition to the clinical examination results, the pathological results and the patient's general condition were used to decide whether to carry out additional treatment, such as radiotherapy and chemotherapy. All of the patients included in the study were operated on by the same team.

### Observation indicators

Events of vascular crisis, flap necrosis and invalid donor trauma were evaluated during the perioperative period. Complications at the donor site were evaluated at the return visit time 6 months after the operation. The evaluation of speech and swallowing functions was conducted at each follow-up visit (1 month, 3 months, 6 months and 1 year after surgery). The primary statistical analysis was conducted at 6 months because healing of the surgical and donor areas was complete at this time, and the speech and swallowing functions were stable.

#### Invalid trauma in the donor area

Due to the variation in perforator vessels and the occurrence of false-positive in the perforator localization examination, if either the perforator could not be found or the diameter of the skin flap did not meet the design during the operation, it was necessary to alter the design of the flap. In addition, in some patients, the tissue in the donor area needed to be clipped when it was closed and sutured. Both of these types of cases were recorded as cases of donor site invalid trauma.

#### Vascular crisis and flap necrosis

The colour and temperature of the flap were closely observed for 24 h after the operation, and vascular exploration was performed in cases of vascular crisis. Cases of flap necrosis were recorded, including cases of partial necrosis and total necrosis. The levels of healing observed in the donor and recipient areas after trauma were recorded.

#### Complications in the donor and recipient areas

Complications that occurred in the donor and recipient areas were recorded at the follow-up. The complications included sensory abnormalities, dyskinesia, circulatory disorders, muscle weakness, scarring, etc.

#### Swallowing function

Swallowing function was evaluated during the postoperative follow-up. The Anderson Dysphagia Inventory (MDADI) [11] was used to evaluate the degree of dysphagia in patients who underwent hemiglossectomy and reconstruction. The MDADI includes 20 questions that address 4 factors: overall state (1 question), emotional state (6 questions), functional state (5 questions) and physical state (8 questions). Each question is scored from 1 to 5 points according to the severity. A lower score corresponds to less severe dysfunction.

#### Language evaluation

The speech function of the reconstructed tongue was evaluated by the speech comprehension score system [12] developed by the MD Anderson Cancer Center. Grades were selected on the basis of the number of pronunciation errors and whether the speech could be understood: 1 represents major errors in pronunciation and an incomprehensible statement; 2 represents multiple errors and a statement that is comprehensible only when the listener understands the topic; 3 represents some errors and a statement that is comprehensible when the listener does not understand the topic; 4 represents few errors and a statment that is comprehensible when the listener does not understand the topic; 5 represents no errors and a fully comprehensible statement.

#### Appearance satisfaction evaluation

Appearance evaluation is part of the fourth edition of the UW-QOL [13] and was selected and slightly modified to evaluate the satisfaction with appearance in the two groups. The satisfaction with appearance was scored as follows: 5 represents no change; 4 represents a slight change; 3 indicates the change in appearance annoys the individual, but they are able to maintain a normal lifestyle; 2 indicates the individual’s appearance is seriously damaged and therefore their lifestyle is limited; 1 indicates the individual’s change in appearance makes them unable to get socialize with others.

### Statistical analysis

SPSS 25.0 was used for data analysis. Descriptive statistical analyses were performed. The count data were expressed as frequencies (percentages). The chi-square test was used for comparisons between the control group and the experimental group. Continuous data are expressed as the mean ± standard error of mean. The independent samples t-test was used for comparisons between the control group and the experimental group for data that had a normal distribution. The rank-sum test was used for comparisons between the two groups for data that did not have a normal distribution.

## Results

### General information

As shown in Table 1, there were no significant differences between the two groups in their general characteristics or T and N stages (P > 0.05). The pathological results showed that all margins were negative, and patients with lymph node metastasis and nerve infiltration underwent adjuvant radiotherapy. There were no significant differences in general information between the two groups (P > 0.05).

**Table 1:** General information and treatment of the patients

	Traditional flap group	L-shaped flap group	P
Sex			
Male	15 (75.0%)	9 (60.0%)	0.344
Female	5 (25.0%)	6 (40.0%)	
Age	54.05 ± 3.10	47.73 ± 3.01	0.163
T stage			
1	5 (25.0%)	2 (13.3%)	0.393
2	15 (75.0%)	13 (86.7%)	
N stage			
0	16 (80.0%)	10 (66.7%)	0.385
1	1 (5.0%)	3 (20.0%)	
2	3 (15.0%)	2 (13.3%)	
Radiotherapy			
No	15 (75.0%)	11 (73.3%)	0.911
Yes	5 (25.5%)	4 (26.7%)	
Flap exploration			
Doppler ultrasonography	5 (25.0%)	0 (0.0%)	0.038
Computed tomography	2 (10.0%)	0 (0.0%)	
Thermal imaging camera	13 (65.0%)	15 (100.0%)	

### Invalid donor trauma and the exploration of perforating branches

The perforating branches in the L-shaped group were well positioned before the operation, and intraoperative replacement was not performed. In the traditional group, intraoperative replacement was performed in one patient, and part of the tissue needed to be repaired in one patient when the donor area was sutured (Table 2).

**Table 2:** Comparison of postoperative conditions of the flap

	Traditional flap group	“L” flap group	P
Vascular crisis/flap necrosis			
Yes	2 (10.0%)	1 (6.7%)	0.727
No	18 (90.0%)	14 (93.3%)	
Invalid donor trauma			
Yes	2 (10.0%)	0 (0.0%)	0.207
No	18 (90.0%)	15 (100.0%)	
Donor complications	2*	–	–

*2 cases of scar hyperplasia (1 case adversely affecting movement)

Before the operation, Doppler ultrasound, computed tomography or a smartphone-compatible thermal imaging camera was used to identify the locations of the blood vessels in the donor area and to locate the surface of the perforating branches in all patients to reduce the operation time and prevent unnecessary injury during the operation (Table 1). During the operation, it was found that the position of the perforating branches was basically consistent with the findings from before the operation, and the design of the flap did not change during the operation for any of the patients. In the traditional group, 5 patients were examined by Doppler ultrasound; 2 patients underwent computed tomographic examinations of the blood vessels of the leg (Table 1), in which most of the positions of the perforating branches during the operation were basically the same as those before the operation; and the donor area of the anterolateral femoral flap was transposed from the left leg to the right leg in only one patient.

### Dysphagia, appearance satisfaction and language evaluation

As shown in Table 3, the degree of global and functional dysphagia of the L-shaped group was less than that of the traditional group, and the difference was statistically significant (P < 0.05). However, there was no significant difference in the emotional or physical scores regarding dysphagia (P > 0.05).

**Table 3:** Comparison of intraoperative and postoperative conditions

	Traditional flap group	“L” flap group	P
Dysphagia			
Global	3.55 ± 0.29	2.60 ± 0.29	0.032
Emotional	16.60 ± 1.36	13.47 ± 1.72	0.113
Functional	15.75 ± 1.22	11.47 ± 1.38	0.032
Physical	26.50 ± 2.30	21.88 ± 2.03	0.107
Language	3.20 ± 0.26	4.13 ± 0.30	0.031
Appearance	3.25 ± 0.23	3.31 ± 0.33	0.498

In the language evaluation, the traditional group had lower scores than the L-shaped group, and the difference was statistically significant (P < 0.05); the L-shaped group had better language recovery during the follow-up.

In the evaluation of appearance satisfaction, there was no difference between the two groups (P > 0.05).

### Recipient and donor complications

Two patients in the traditional group and 1 patient in the L-shaped group had partial necrosis of the flap after the operation, but the cases healed well after debridement and dressing changes. After 4 to 6 months of follow-up, it was found that in the traditional group, 1 patient had slight scar hyperplasia in the donor area that did not affect sensation or function, 1 patient had obvious scar hyperplasia in the donor and recipient areas, and the case in the donor area had a slight influence on motor function due to scar contracture (Table 2).

### Case presentation

**Fig. 3:**
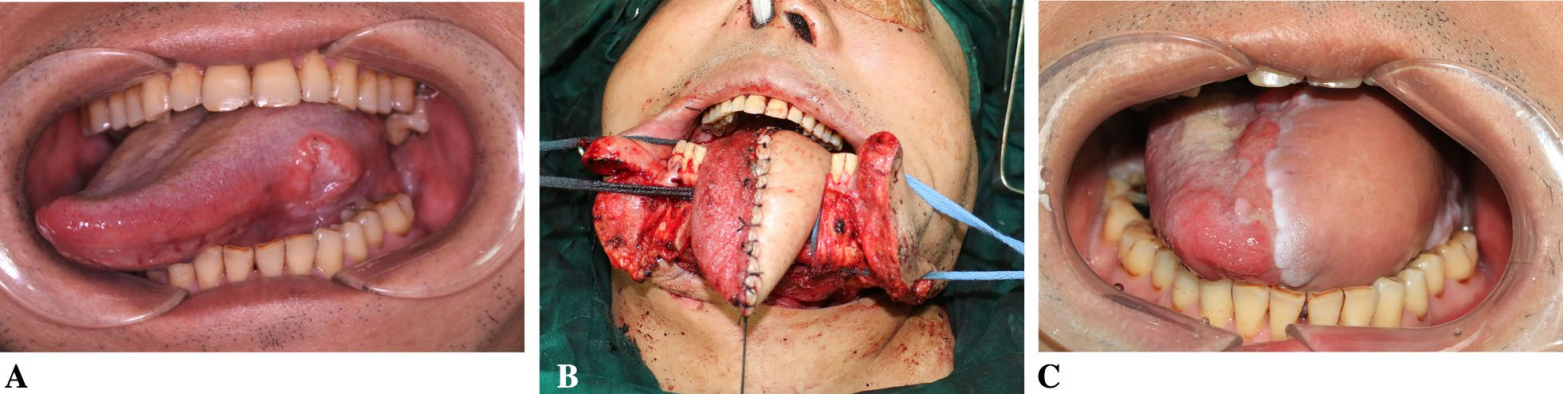
Hemiglossal defect repair with the L-shaped AlTP. **A** The tumour was located in the posterior 1/3 of the left side of the tongue; **B** after the tumour was removed, L-shaped AlTP was used to repair the tongue; **C** half a year after the operation, the patients' lingual motor function was good

Figure 3 shows a case where the tumour occurred on the left side of the tongue, and it was treated with tongue tip-preserving hemiglossectomy. After resection, an L-shaped ALTP was used for repair, and reconstruction of the tongue defect was successful. Six months after the operation, the patients' tongue motor function was good (Fig. 3, Additional file 4: Fig. S3, Additional file 5: Fig. S4).

**Fig. 4:**
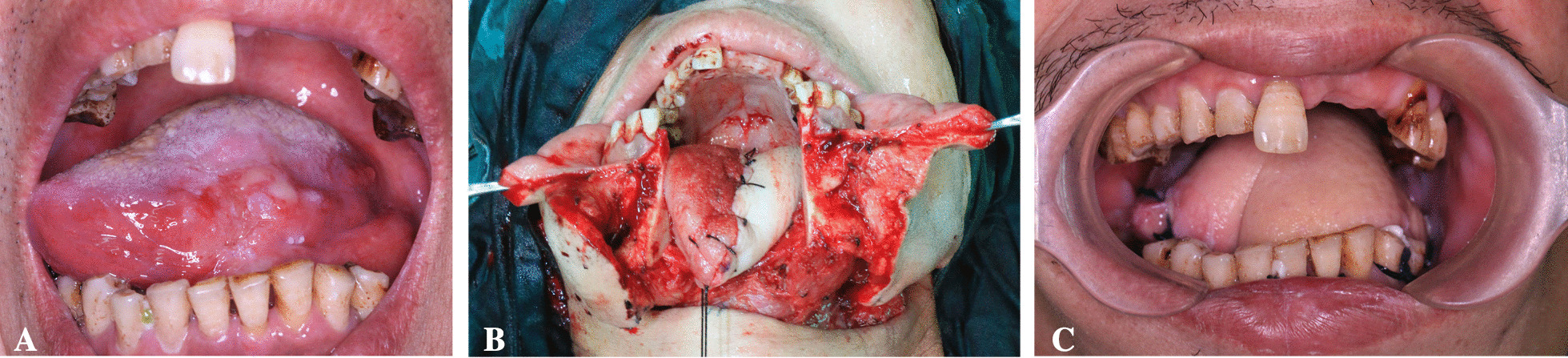
Hemiglossal defect repair with traditional AlTP. **A** The tumour was located in the middle 1/3 of the left side of the tongue; **B** traditional AlTP was used to repair the tumour; **C** One month after the operation, the flap bulged and occupied the space, with the tip of the tongue pinned to the right

The tumour shown in Fig. 4 occurred in the middle of the left side of the tongue and was treated with standard hemiglossectomy. A traditional ALTP was used to reconstruct the tongue defect. The tongue’s shape was restored immediately after the operation. One month after the operation, the flap bulged and occupied space, pushing the tip of the tongue towards the healthy side (Additional file 6: Fig. S5).

## Discussion

The tongue has various and complex functions that play important roles in swallowing and speech.

With the development of functional microsurgery, various pedicled tissue flaps have been used to repair and reconstruct the shape and function of the tongue. These flaps were initially developed to simply fill the dead cavity and close the wound to maximize the recovery of various functions of the tongue.

In 1984, Song [14] reported the first clinical application of ALTP. ALTP has many advantages: it has a long vascular pedicle, it remains in the same position, it only slightly affects the function of the donor area, and it can be taken from a donor area that can provide a large amount of tissue. It can repair a large tongue defect combined with a pharyngeal defect. It has a large vascular diameter and is easy to anastomose; the distance between the donor area and the recipient area is large, and the operation can be carried out in two areas at the same time. The wound at the donor area can be directly sutured, and its location can be hidden. The branches of the lateral femoral cutaneous nerve and the motor nerve of the flap are left intact, so the sensory and motor nerves of the tongue can be anastomosed after transplantation, which is helpful to restore the sensory function of the flap and increase the ability of the reconstructed tongue to move.

**Fig. 5:**
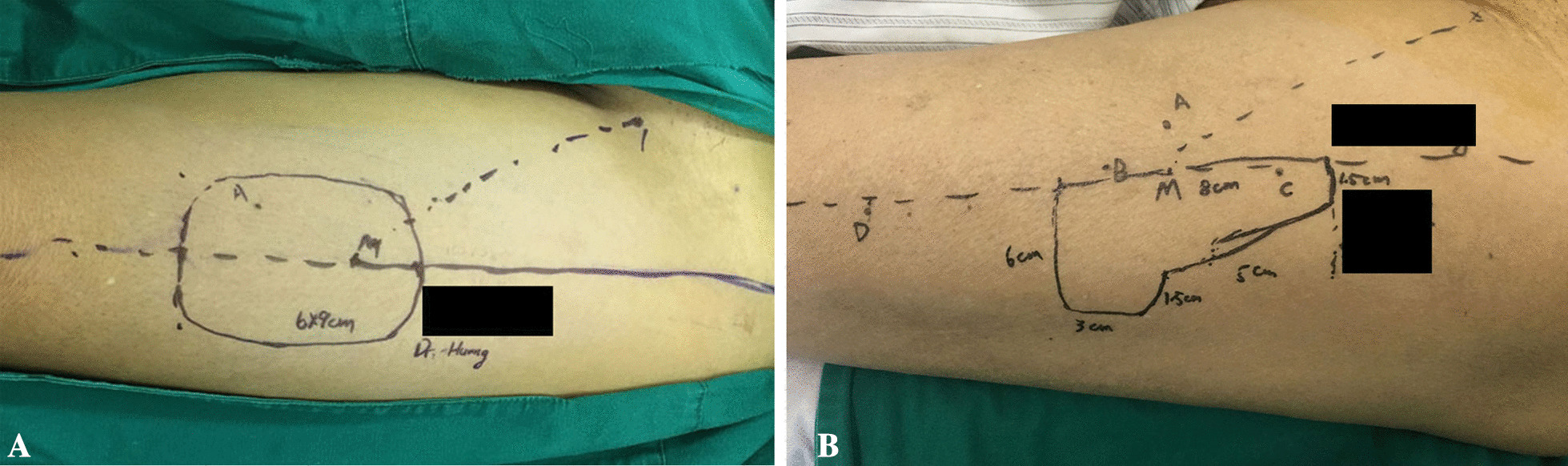
Two kinds of L-shaped ALTP preparation. **A** Traditional ALTP design. **B** L-shaped flap design

However, ALTP also has some limitations; for example, it can lead to skin flap necrosis, infection, unsatisfactory postoperative function and appearance. To reduce the occurrence of complications and obtain better postoperative reconstruction results, previous studies have proposed various approaches to tongue reconstruction, but their design is complex, making them difficult to apply in clinical practice [15]. As shown in Fig. 5, we aimed to design a new type of ALTP flap that ensures functional and morphological recovery in patients with half-tongue defects, has the simplest design possible, and is easy to use. Both flaps showed good performance immediately after tongue reconstruction, but in long-term follow-up, traditional ALTP showed hypertrophy of the tongue and this affected the tongue function (Fig. 4), which did not seem to occur in the L-shaped flap group (Fig. 3).

Based on the extensive and favourable characteristics of the ALTP, we designed an L-shaped flap that was modified according to the shape of the half-tongue defect undergoing repair, making the best use of the tissue in the donor area after radical operations for tongue cancer and achieving good recovery of the function of the tongue while preserving as much of the tissue in the donor area as possible.

Sufficient tissue volume is very important during tongue reconstruction to avoid restricting the activity of the remaining tissue in the body of the tongue. Preservation of the donor site tissue and avoidance of invalid trauma are also important for the function and appearance of the donor sites. Currently, there are many methods used to locate the perforators, such as Doppler ultrasound, CTA, and MRA [16–19]. Because of the high cost and long scanning time required for MRA, we used three other methods to locate the perforator. A smartphone-compatible thermal imaging camera [10] was used in the new L-type flap group, Doppler ultrasound was used in 5 patients, and CT was used in 2 patients in the traditional flap group. Almost all of the positions of the perforating branches in the patients were consistent with the preoperative scan positions, except for one patient in the traditional group for whom a replacement of the perforator was not found during the operation. In addition, there were also patients who needed to undergo tissue clipping before donor suturing. We found that there was no significant difference between the infrared method and other methods in terms of the operation time and the accuracy in finding perforating branches Intraoperatively. Except for one patient who required a changed in the position of the flap, all of the flaps were obtained within 30 to 40 min.

In the L-shaped group, the recovery of the donor site was better than that of the traditional flap group. We sutured points E and B in pairs, and the rest of the points were sutured in sequence with the new pairs. This approach reduced the pressure required during placing the sutures, and the L-shaped design reduced the need for trimming the tissue before stitching. In our study, there were no cases of donor site invalid trauma in the L-shaped flap group. Hence, the L-shaped design was conducive to restoration of the donor site. In the traditional group, two patients had scar hyperplasia in the donor area, one of which affected leg movement. In contrast, 15 patients did not have invalid wounds in the donor area, and no patients had scar hyperplasia or other complications, such as motor and sensory impairment, at the postoperative follow-up.

The L-shaped defects in the donor area were easily closed and sutured. Inaddition to improvements at the donor area, our main innovation is in the flap design, which was selected according to the shape of the half-tongue defect. Compared with the traditional flap that adopts the shape of a circle and square, this flap has an L-shaped design, is better shaped to rebuild the tongue frenum, and has less adhesion and more mobility. In some patients who underwent traditional flap repair, the effect of the immediate repair was acceptable. However, because the flap shape was not completely aligned with the defect shape, it was found that the tip of the tongue was pushed towards the healthy side by the hypertrophic flap, the range of movement was limited, and the vocal and swallowing functions were affected.

The tip of the tongue is lined with fungous papillae containing taste buds that can detect sweetness, and the tip of the tongue is important for the pronunciation of certain words. Therefore, for tumours located in the middle and posterior lingual margins, we preserved the tongue tip in the safe area during the resection. After follow-up of the L-flap group, We found that there was no significant difference in the impact on taste, swallowing or speech between the two groups (P > 0.05), but it should be noted that all of the patients in the tip-preserving group retained their ability to taste on the tip of the tongue, while 3 patients with standard hemilingual resection had partial or complete loss of taste at the tip of the tongue (Additional file 7: Supplemental Table S4).

The MDADI was used to evaluate postoperative swallowing function [20–24]. The results show that the L-shaped group was superior to the traditional group in terms of dysphagia and language function.

In summary, the L-shaped flap can maximize the use of flap tissue to treat tongue defects, and L-shaped flap repair is associated with fewer complications in the donor and recipient areas and better postoperative recovery of swallowing and language functions than traditional flap repair. Due to the small number of cases in this study, more studies need to be conducted to verify the findings. In future work, we will increase the sample size and carry out additional research to improve postoperative functional recovery and reduce the occurrence of complications.

## Supplementary Information

**Additional file 1: Video S1.** The perforating branch in the donor area was examined using Doppler ultrasound or a smartphone-compatible thermal imaging camera

**Additional file 2: Figure S1.** The entire process of preparing the L-shapedALTP. A. A and B. The perforating branches were examined. C. According tothe perforator, an L-shaped flap was designed, and at least one perforatorwas retained. D. The flap was prepared. E. After the flap was removed, themuscular sleeve was sutured. F. The donor area of the thigh was sutured,and a drainage tube was left in place

**Additional file 3: Figure S2.** The entire process of preparing the L-shapedALTP. A. Based on the perforators, an L-shaped flap was designed, andat least one perforator was retained. B. After the flap was obtained, theperforating branches were examined. C. The flap was prepared. D. Afterthe flap was removed, the muscular sleeve was sutured. E. The donor areaof the thigh was sutured, and a drainage tube was left.

**Additional file 4: Figure S3.** The entire process of hemiglossal defectrepair with the L-shaped AlTP. A. The tumour was located in the posterior1/3 of the left side of the tongue; B. After the mandible was opened, thetumour was fully exposed; C. After the tumour was removed, L-shapedAlTP was used to repair the tumour; D. The shape of the tongue wasgood at 2 weeks after the operation; E. Half a year after the operation, thepatients’ lingual motor function was good; F. Half a year after the operation,the lateral movement of the patient’s tongue was good.

**Additional file 5: Figure S4.** The entire process of the hemiglossal defectrepair with L-shaped AlTP. A. The tumour was located in the posterior 1/3of the right side of the tongue; B. After the mandible was opened, thetumour was fully exposed; C and E. Tongue defect after standard hemiglossectomy;D. Specimens after tumour resection; F and G. L-shaped AlTP was used to repair the tumour; H and I. After the defect was repaired withthe L-shaped flap, the appearance of the reconstructed tongue was good.

**Additional file 6: Figure S5.** The entire process of hemiglossal defectrepair with traditional AlTP. A. The tumour was located in the middle 1/3 ofthe left side of the tongue; B. After the mandible was opened, the tumourwas fully exposed; C. Tongue defect after removal of the tumour; D. Thetraditional anterolateral perforator flap was made; E. The perforatingbranches showed that the blood vessels of the flap were sufficiently long;F. The traditional AlTP was used to repair the tumour, and the shape of thetongue was good; G. One month after the operation, the flap bulged andoccupied the space, and the tip of the tongue was directed towards theright.

**Additional file 7: Table S4.** Odds Ratios of IgM or IgG or PCR positive HCWs
