# Supplementary figures and images for: Application of an L-shaped anterolateral thigh flap in reconstruction after hemiglossectomy
Source: BMC Surg. 2022 Jan 29;22:32. doi: 10.1186/s12893-022-01473-7 (PMC8800230; doi:10.1186/s12893-022-01473-7)

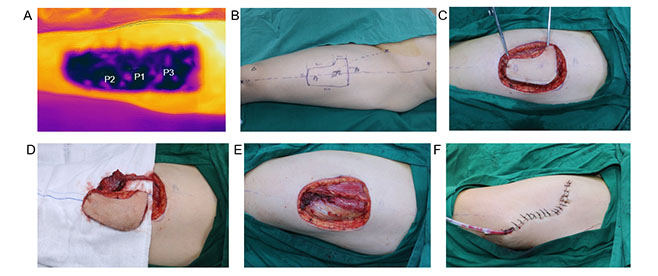

Supplement: Supplementary file 2 — Additional file 2: Figure S1. The entire process of preparing the L-shapedALTP. A. A and B. The perforating branches were examined. C. According tothe perforator, an L-shaped flap was designed, and at least one perforatorwas retained. D. The flap was prepared. E. After the flap was removed, themuscular sleeve was sutured. F. The donor area of the thigh was sutured,and a drainage tube was left in place [file 12893_2022_1473_MOESM2_ESM.jpg]

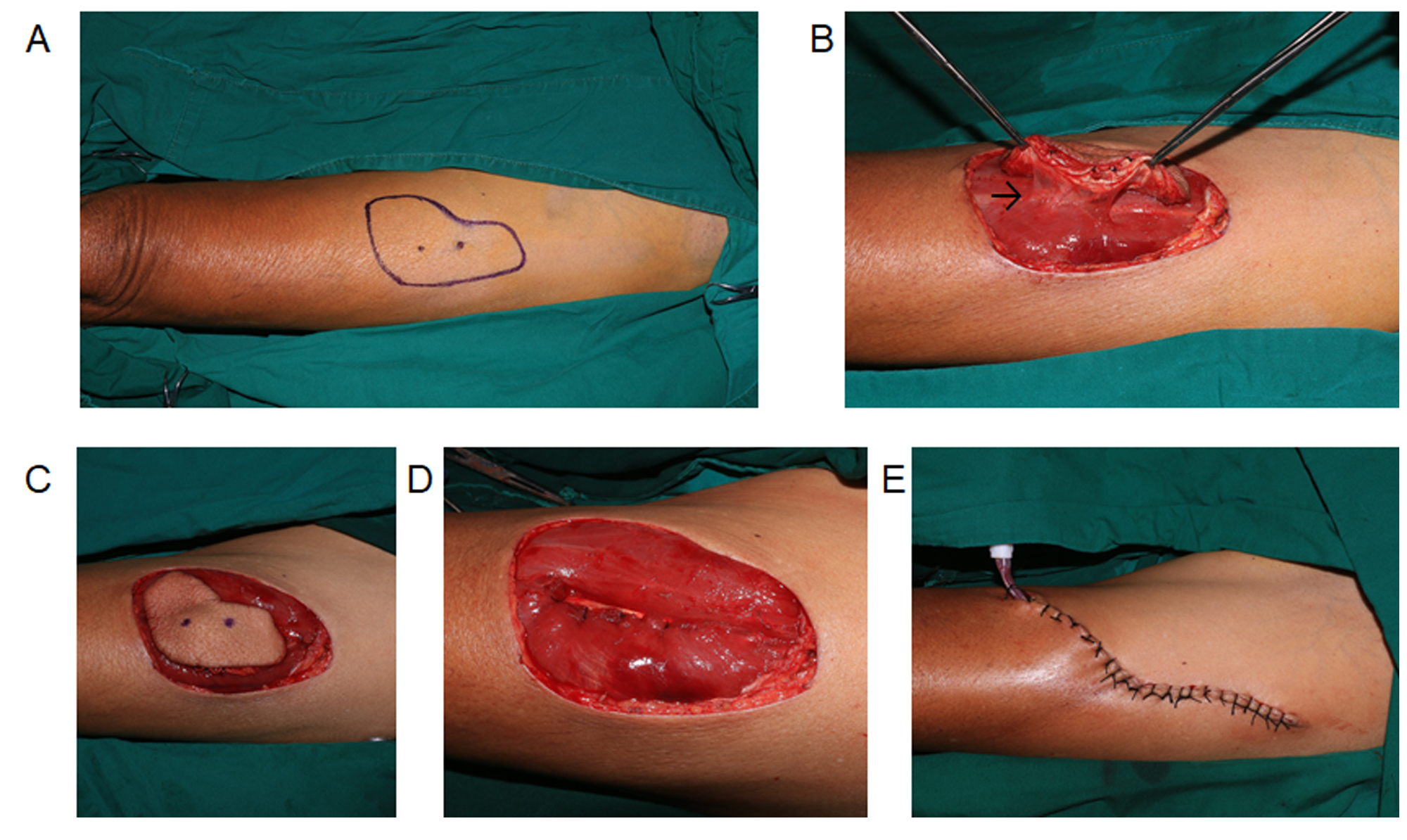

Supplement: Supplementary file 3 — Additional file 3: Figure S2. The entire process of preparing the L-shapedALTP. A. Based on the perforators, an L-shaped flap was designed, andat least one perforator was retained. B. After the flap was obtained, theperforating branches were examined. C. The flap was prepared. D. Afterthe flap was removed, the muscular sleeve was sutured. E. The donor areaof the thigh was sutured, and a drainage tube was left. [file 12893_2022_1473_MOESM3_ESM.jpg]

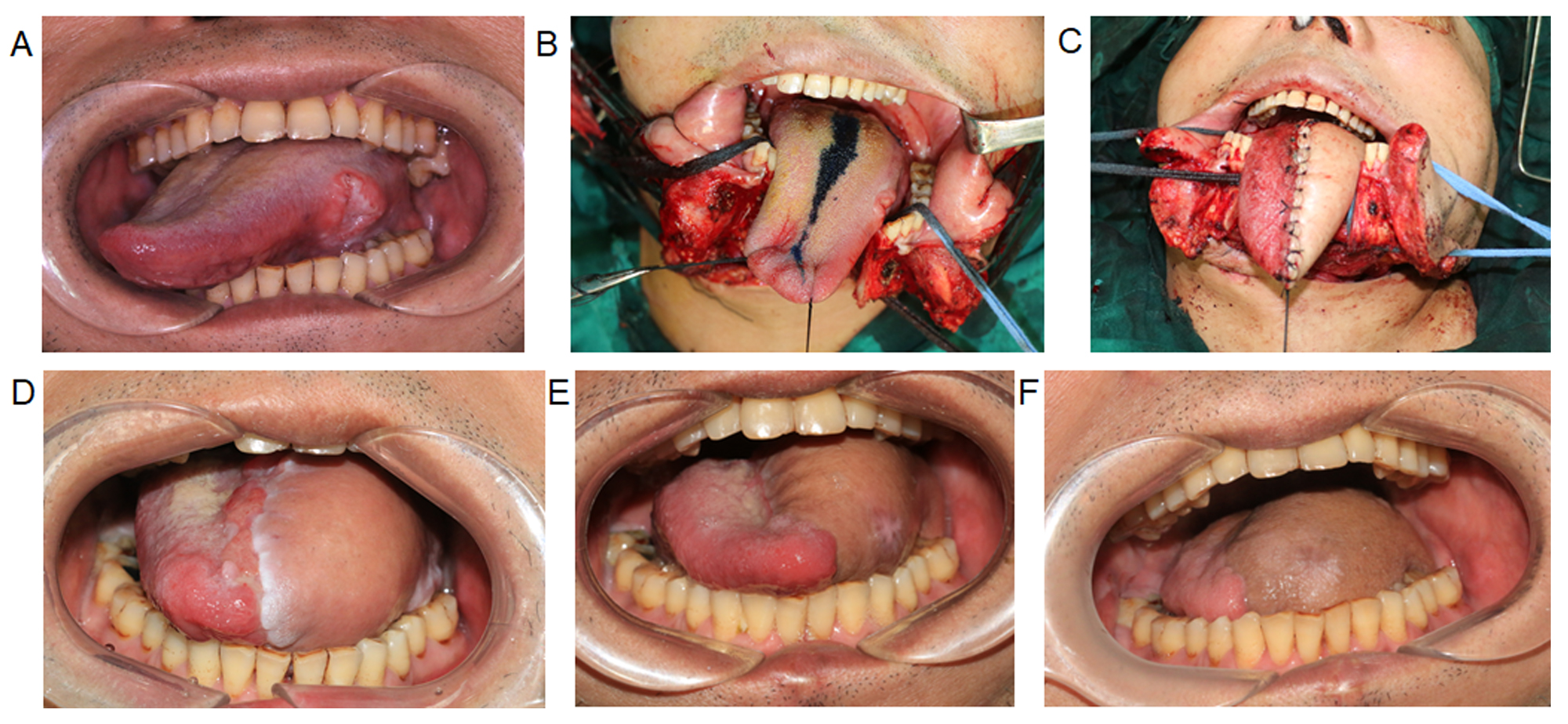

Supplement: Supplementary file 4 — Additional file 4: Figure S3. The entire process of hemiglossal defectrepair with the L-shaped AlTP. A. The tumour was located in the posterior1/3 of the left side of the tongue; B. After the mandible was opened, thetumour was fully exposed; C. After the tumour was removed, L-shapedAlTP was used to repair the tumour; D. The shape of the tongue wasgood at 2 weeks after the operation; E. Half a year after the operation, thepatients’ lingual motor function was good; F. Half a year after the operation,the lateral movement of the patient’s tongue was good. [file 12893_2022_1473_MOESM4_ESM.jpg]

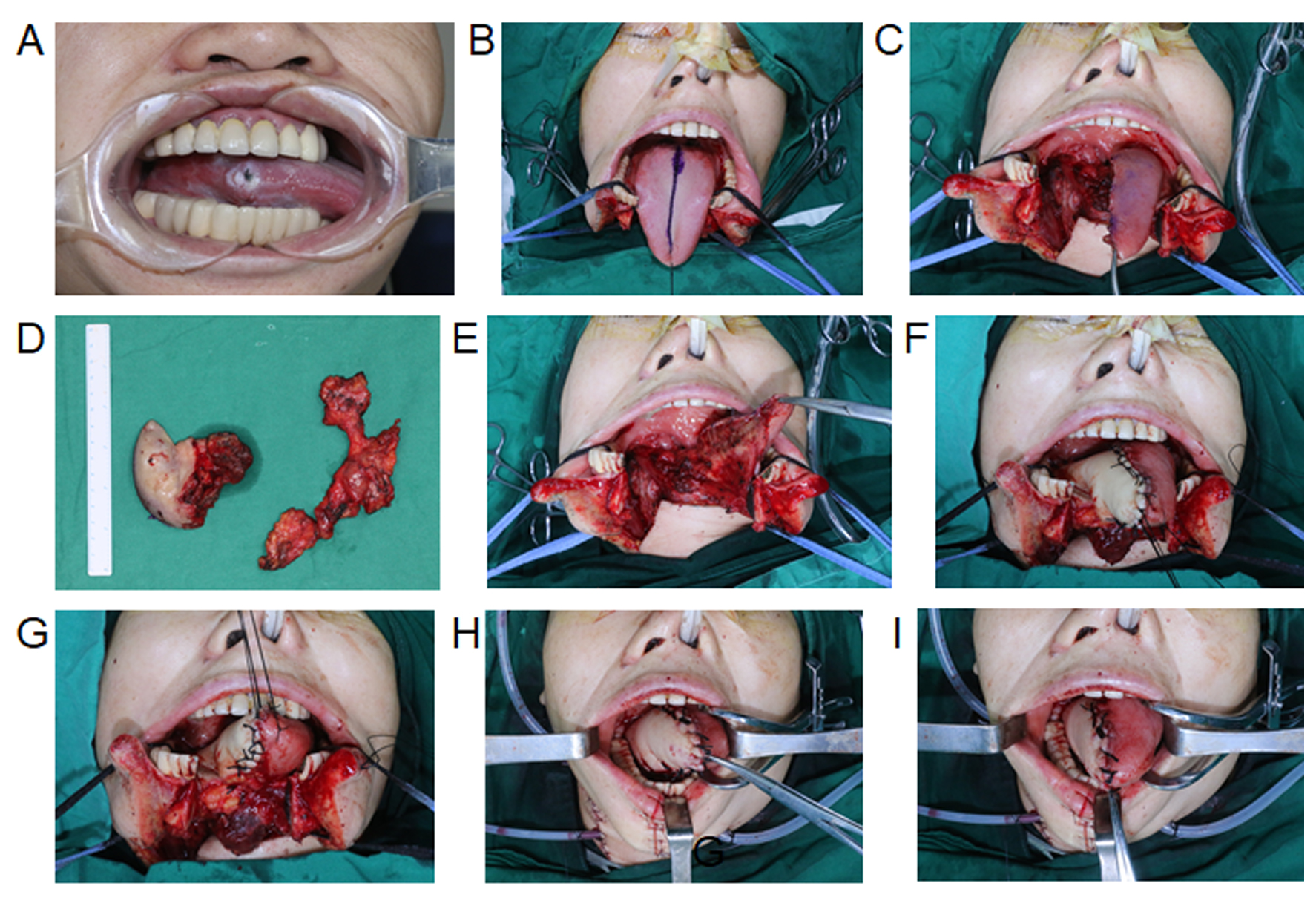

Supplement: Supplementary file 5 — Additional file 5: Figure S4. The entire process of the hemiglossal defectrepair with L-shaped AlTP. A. The tumour was located in the posterior 1/3of the right side of the tongue; B. After the mandible was opened, thetumour was fully exposed; C and E. Tongue defect after standard hemiglossectomy;D. Specimens after tumour resection; F and G. L-shaped AlTP was used to repair the tumour; H and I. After the defect was repaired withthe L-shaped flap, the appearance of the reconstructed tongue was good. [file 12893_2022_1473_MOESM5_ESM.jpg]

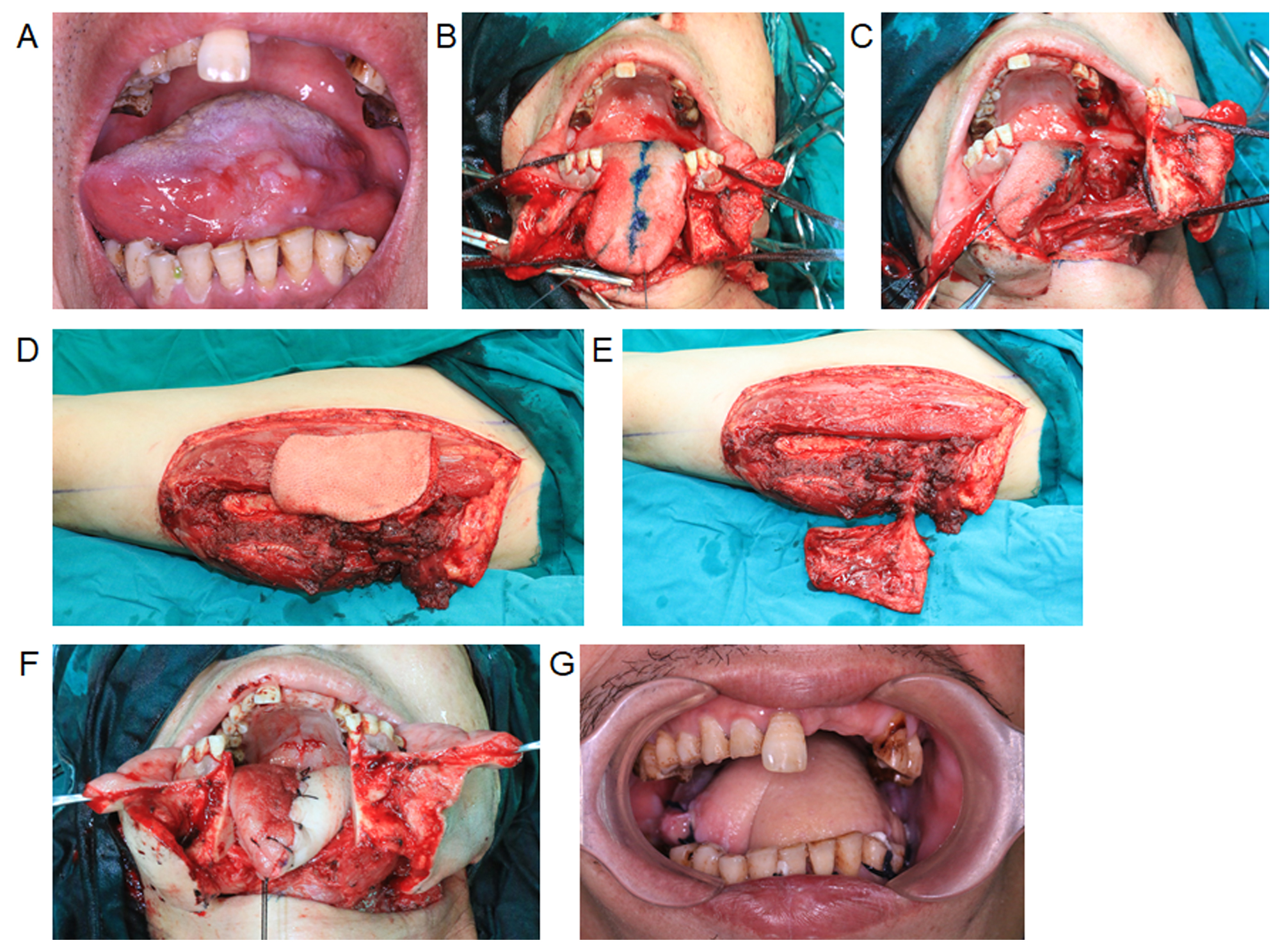

Supplement: Supplementary file 6 — Additional file 6: Figure S5. The entire process of hemiglossal defectrepair with traditional AlTP. A. The tumour was located in the middle 1/3 ofthe left side of the tongue; B. After the mandible was opened, the tumourwas fully exposed; C. Tongue defect after removal of the tumour; D. Thetraditional anterolateral perforator flap was made; E. The perforatingbranches showed that the blood vessels of the flap were sufficiently long;F. The traditional AlTP was used to repair the tumour, and the shape of thetongue was good; G. One month after the operation, the flap bulged andoccupied the space, and the tip of the tongue was directed towards theright. [file 12893_2022_1473_MOESM6_ESM.jpg]
