# Supplementary material for: Application of an L-shaped anterolateral thigh flap in reconstruction after hemiglossectomy
Source: BMC Surg. 2022 Jan 29;22:32. doi: 10.1186/s12893-022-01473-7 (PMC8800230; doi:10.1186/s12893-022-01473-7)
Supplement: Supplementary file 7 — Additional file 7: Table S4. Odds Ratios of IgM or IgG or PCR positive HCWs [file 12893_2022_1473_MOESM7_ESM.docx]

**Tables**

Supplemental Table 4 Comparison of Intraoperative and Postoperative Conditions from the “L” Flap Group

|  | Standard hemiglossectomy | Tongue tip preserving hemiglossectomy | P |
| --- | --- | --- | --- |
| Sense of taste |  |  |  |
| Yes | 6 (66.7%) | 6 (100.0%) | 0.114 |
| No | 3 (33.3%) | 0 (0.0%) |  |
| Dysphagia |  |  |  |
| Global | 2.22 ± 0.32 | 3.17 ± 0.48 | 0.112 |
| Emotional | 13.78 ± 2.27 | 13.00 ± 2.86 | 0.834 |
| Functional | 11.56 ± 1.88 | 11.33 ± 2.19 | 0.940 |
| Physical | 20.00 ± 3.13 | 24.33 ± 2.66 | 0.345 |
| Language | 4.00 ± 0.24 | 4.00 ± 0.26 | 0.999 |
| Appearance | 3.44 ± 0.41 | 3.33 ± 0.61 | 0.878 |
